# Evidence of the effect of primary care expansion on hospitalizations: Panel analysis of 143 municipalities in the Brazilian Amazon

**DOI:** 10.1371/journal.pone.0248823

**Published:** 2021-04-08

**Authors:** Vânia Cristina Campelo Barroso Carneiro, Paulo de Tarso Ribeiro de Oliveira, Saul Rassy Carneiro, Marinalva Cardoso Maciel, Janari da Silva Pedroso

**Affiliations:** 1 Postgraduate Program in Psychology, Federal University of Pará, Belém, Pará, Brazil; 2 João de Barros Barreto University Hospital, Federal University of Pará, Belém, Pará, Brazil; 3 Faculty of Statistics, Federal University of Pará, Belém, Pará, Brazil; University of Western Australia, AUSTRALIA

## Abstract

**Background:**

The Family Health Strategy (FHS) became consolidated as a primary care model and gatekeeper for the Unified Health System (Sistema Único de Saúde, SUS) in the Brazil and it is considered one of the largest primary health care programmes in the world. Its rapid expansion allowed the SUS to meet the changing health care needs of the population remote localities of Brazilian municipalities.

**Methods:**

In the present study, exploratory data analysis was performed using modelling to provide a general overview of the study and to delineate possible structural characteristics of the cross-sectional time-series data. Panel regression methods were used to assess the association between FHS coverage and ambulatory care-sensitive hospitalizations (ACSH rates) in the municipalities of Pará, in the Brazilian Amazon, from 2008 to 2017.

**Results:**

The results showed strong evidence for the association between FHS coverage and ACSH rates, including reductions of 22% in preventable hospitalizations and 15% in hospital expenses that were directly linked to the 40% increase in FHS population coverage during the evaluated period. This expansion of primary care has mainly benefitted areas that are difficult to access and populations that were previously deprived of health care in the vast Amazon territory.

**Conclusions:**

The findings of this study show that the increase of the expansion of primary care reduces the preventable hospitalization and the hospital expenses. This reinforces the need for public protection of the health of populations at risk and the positive impacts of primary care in the Brazilian Amazon.

## Introduction

Brazil has a public health system called the Unified Health System (Sistema Único de Saúde, SUS) that meets the principles of universal and comprehensive service coverage [1]. This care model is based on socio-environmental aspects and on the understanding that health is a right of the citizen and duty of the State [2, 3]. The SUS Family Health Strategy (FHS), the system’s primary health care model, provides care through multidisciplinary teams in a geographically defined territory for its corresponding population and comprises the patient’s first contact with the health system [4, 5]. The FHS is considered one of the largest primary health care programmes in the world; it served 64% of the Brazilian population (127 million people) in 2014 [6, 7].

Despite the expansion of the FHS in recent years, the distribution of teams in Brazilian municipalities is heterogeneous because of high physician turnover, especially in remote areas and vulnerable locations in the North region of the country [8–10]. To address common complaints about physician shortages and limited access to primary care services in municipalities, the More Doctors Programme (Programa Mais Médicos, PMM) was created in 2013, in partnership with the Pan American Health Organization. Its main objectives include the placement of Brazilian and foreign physicians in rural territories and on the outskirts of large Brazilian cities to ensure the population’s right to health, with primary care providers serving as the gatekeepers of the system [11–13].

The North region of Brazil consists of the vast Amazon territory, and its most populous state is Pará. This state is one of the most valuable geographical spaces on the planet due to its complex biodiversity and because it concentrates one-third of the Amazon forest, the largest hydrographic basin and the most complex mineral province in the world. With a substantial isolated rural population on riverbanks, Pará is unique in the world, and its municipalities have the lowest human development indicators in the country [14–16]. A significant number of municipalities in Pará were served by the PMM in 2013 and sought medical care through primary care services.

In this study, we examine the association between primary care coverage in the FHS and ambulatory care-sensitive hospitalizations (ACSHs) between 2008 and 2017, adjusting for socioeconomic and demographic variables, in municipalities of the state of Pará in the Brazilian Amazon. ACSH is an indicator used worldwide to assess the accessibility and effectiveness of primary health care [17, 18]. The recent expansion of the FHS in Pará functioned as a natural experiment because the expansion of primary health care was rapid, but the implementation varied considerably among the municipalities of the state.

## Materials and methods

### Data source

The expansion of the FHS, represented by the expansion of primary care coverage, was explored to evaluate its possible effect on changes in preventable hospitalizations and ACSHs in the municipalities of the state of Pará. In this longitudinal analysis from 2008 to 2017, data were collected from the Brazilian Ministry of Health via the DATASUS website, and population estimates were collected from the Brazilian Institute of Geography and Statistics (Instituto Brasileiro de Geografia e Estatística, IBGE). This information was used to determine values for the study variables: FHS population coverage, ACSHs, hospitalization expenses and Municipal Health Fund. Data from 143 municipalities in the state of Pará were analysed; one municipality was not represented in the sample because this municipality only attained administrative autonomy in 2013 and complete data necessary for the analysis were not available for this municipality.

The data used are in the public domain and do not require approval in ethics and research committees according to Brazilian law because they do not directly involve people and medical reports, being only a research with databases, whose information is aggregated, without the possibility of individual identification [19].

The FHS is a national health programme guided by the principles of primary care that functions as a gatekeeper to SUS health services in Brazilian municipalities and follows uniform care protocols in all Brazilian states [20]. Population coverage by primary health care was defined as the number of implemented FHS teams (comprising a doctor, a nurse and three community health workers assigned to a population of 3000 people residing in a given territory, according to the Brazilian Ministry of Health).

Population coverage in the state of Pará was categorized as high (greater than 70%), intermediate (between 50 and 70%) and low (less than 50%). Ideally, the number of FHS teams would be sufficient to cover 100% of a municipality’s population [21]. The study period was based on recent FHS milestones considering the national health policy.

The municipality was chosen as the unit of study because it is the administrative level responsible for implementing the FHS and the lowest level for which there are available data on exposure and results. Additionally, to control for spatial heterogeneity, municipalities were aggregated into geographic mesoregions according to the IBGE classification [22] as follows: Lower Amazon, Marajó, Metropolitan Belém, Northeast Pará, Southeast Pará and Southwest Pará. This grouping allowed the evaluation of the possible impacts of population coverage on preventable hospitalizations in the state.

The ACSH rate aims to monitor the reduction in hospitalizations for ambulatory care-sensitive conditions and has been estimated in Brazil since 2008 by an ordinance published by the Ministry of Health [23]. This is a set of diagnoses, based on the International Classification of Diseases (ICD-10), for which effective primary care would reduce the number of hospitalizations by providing preventive measures, early diagnosis of diseases, timely treatment of acute pathologies and control and monitoring of chronic pathologies [24].

The crude ACSH rates were calculated using spreadsheets and data on hospital morbidity and the population exposed to risk in each municipality studied. The main data source was the Hospital Information System of the SUS (SIH-SUS/DATASUS), which provided AIH-1 (hospital admission authorization) files from which information was extracted on hospitalizations in 143 municipalities in the state of Pará, Brazil, calculated per 10,000 inhabitants and compared with the non-ACSH rates in the selected geographic units [25]. The reading of the microdata from the AIHs considered 12 monthly files per year for each of the 143 municipalities from 2008 to 2017, totalling 4,939,017 hospitalizations in the state of Pará, of which 28% (1,376,142 hospitalizations) were ACSHs.

These data were organized in two ways for analysis: i) first, the data were aggregated by municipality and year to obtain information on the number of hospitalizations and the cost of ACSHs, generating a panel of 143 municipalities for the period from 2008 to 2017; ii) second, the data were first separated by disease group according to the 19 disease groups considered in the Brazilian legislation; subsequently, the data were aggregated by municipality and year to obtain the number of hospitalizations for the 143 municipalities for each disease group.

In the general database, the variable "cost of hospitalization per capita" was generated using the ratio of the cost of hospitalization to the number of hospitalizations in each municipality for the period studied. After calculating these costs, the percentage variation in the ratio of the cost of hospitalization to the number of hospitalizations in each municipality was determined, in addition to the total cost of hospitalizations in the state of Pará. Deflation techniques were applied according to the consumer price index (índice de preços ao consumidor amplo [IPCA] in Portuguese, measured by the IBGE) to evaluate the inflationary differences over the years of study. The secondary outcomes, used to control for the socioeconomic heterogeneity among the municipalities of Pará, included information on the Municipal Health Fund (MHF), a transfer of financial resources for health from the federal government to the municipalities for use for the FHS and local hospitals.

### Statistical analysis

Exploratory data analysis was performed using modelling to provide a general overview of the study and to delineate possible structural characteristics of the cross-sectional time-series data. Panel regression methods were used to assess the association between FHS coverage and ACSH rates in the municipalities of Pará from 2008 to 2017. The regression model for the panel data was defined as follows:

$$y_{it}=X_{it}\beta +\varepsilon_{it},$$

*y*_*it*_ is the value of the dependent variable for sampling unit *i* at time *t*,

***X***_*it*_ is the matrix of explanatory variables for sampling unit *i* at time *t*,

***β*** is the vector of the parameters,

*ε*_*it*_ are the composite errors.

The errors represent the unobservable effects, and the following structure was assumed in the panel structure to specify these errors:

$$\varepsilon_{it}=\alpha_{i}+\eta_{it},$$

where α_i_ is time constant unobserved heterogeneity and *η*_*it*_ is the idiosyncratic error, or time-varying unobserved heterogeneity.

The fixed effects model was used to control the unobserved heterogeneity that vary between individuals but that are constant over time. The Hausman test rejects the null hypothesis that random estimation provides consistent estimates so the fixed effects estimator was most suitable for the sample considering the correlation of the intercept with the explanatory variables [26]. The estimations were performed for the dependent variables "rate of ACSH" and "cost of ACSH". For each dependent variable, three models were estimated:

Model 1: Only "FHS coverage" was used as an explanatory variable;

Model 2: In addition to "FHS coverage", variables were added to control for socioeconomic heterogeneity and the MHF indicator;

Model 3: Indicator variables of the mesoregion to which the municipality belongs were added to model 2 to capture some spatial heterogeneity.

Several additional analyses were performed to evaluate the effect of the FHS on hospital admissions, and the estimates with the corresponding 95% CIs were plotted. Subsequently, the data were further explored to determine whether preventable hospital admissions were influenced by the expansion of FHS coverage in the municipalities in order to increase the robustness of the results. The main assumption was that changes in FHS coverage are correlated with hospital admissions and that other factors, such as socioeconomic factors, financial investments in health by the federal government (MHF) and the costs of preventable hospitalizations, could affect this association.

For the panel data analysis technique, after a difference was detected among the mesoregions in the descriptive analysis, it was decided to deepen the analysis by including variables related to the geographical locations of the municipalities, considering the potential migration of individuals across borders to seek hospital care. The sensitivity of the results to the inclusion of additional covariates and alternative estimation models was tested as a standard diagnosis for bias in these models [27].

The cartographic bases and digital networks used in the georeferencing maps were obtained from the public domain of IBGE website [28]. The maps was carried out, with the help of Qgis 2.18 software.

## Results

**Fig 1:**
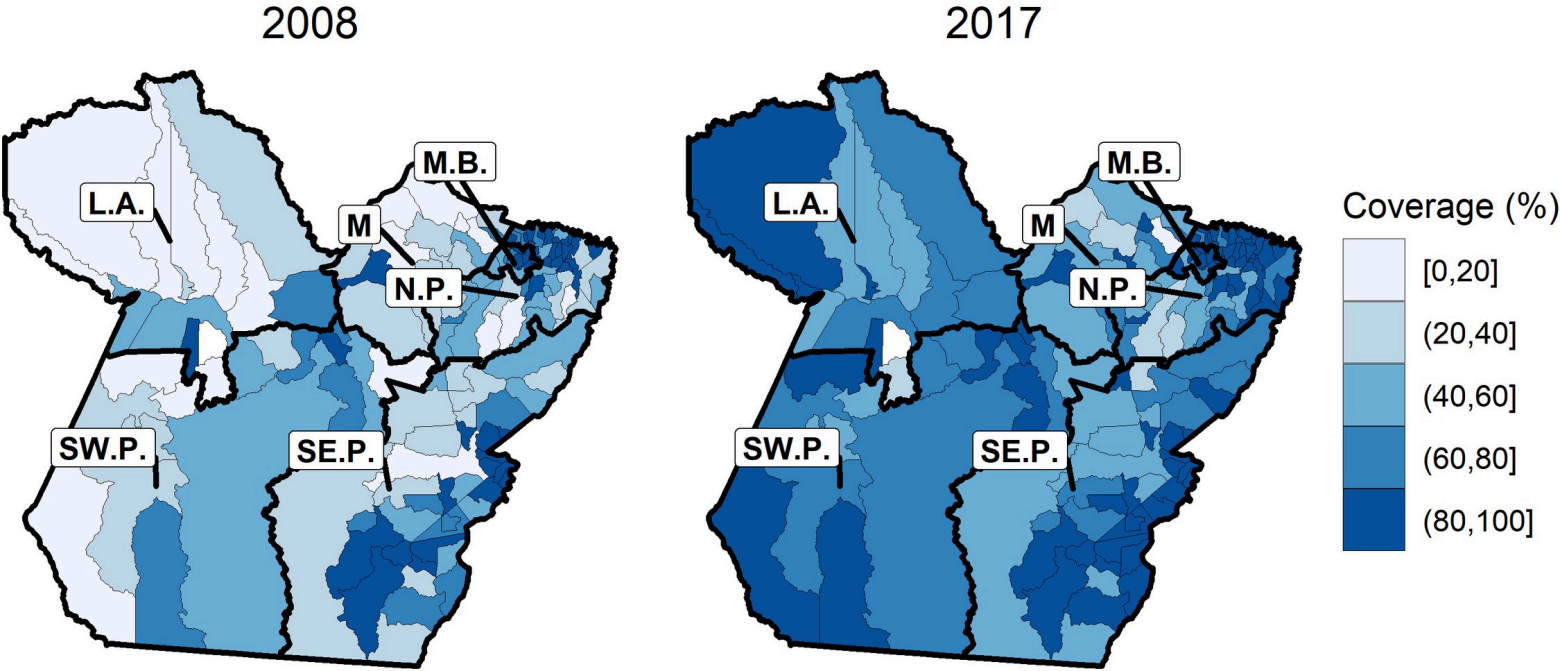
Primary care coverage in the state of Pará, 2008–2017. Municipal distribution of the typology considering the expansion of health coverage in the municipalities of the State of Pará, the lighter colors represent lower coverage and the darker colors mean an increase in health coverage. Source: Own elaboration on maps by Brazilian Institute of Geography and Statistics (IBGE).

The population coverage of the FHS in the state of Pará increased significantly during the evaluated period, from 56% (440 teams) in 2008 to 78% (656 teams) in 2017. Fig 1 shows that during this period, the population coverage increased by 40% in all mesoregions of the state.

In the comparison among the mesoregions (Fig 1), there was a more significant increase in coverage in the Lower Amazon (from 30% to 70%), Southwest Pará (from 40% to 79%) and Marajó (from 29% to 59%) mesoregions. Marajó remained the mesoregion with the lowest population coverage in the state throughout the study period.

Table 1 presents the comparative analysis of 2008 and 2017 by mesoregion and coverage class, which revealed a 71% reduction in the number of municipalities in the low coverage class (less than 50% coverage) and an increase of approximately 80% in the number of municipalities in the high coverage class (greater than 70% coverage). It is noteworthy that in 2008, of the 16 municipalities that make up the Marajó mesoregion, 14 were allocated to the low coverage class, and in 2017, only four municipalities were still in this coverage class.

**Table 1:** Rate of ACSH by primary care population coverage class and mesoregions of the state of Pará, 2008–2017.

2008	Coverage class		
	1-Less than 50%	2-Between 50% and 80%	3-More than 80%
Lower Amazon	167.30 (142.28)	301.09 (122.22)	185.06*
Marajó	138.78 (98.98)	244.71*	21.68*
Metropolitan Belém	329.83 (324.37)	133.65 (48.11)	331.823 (264.31)
Northeast Pará	247.74 (121.92)	171.42 (112.46)	131.56 (98.00)
Southeast Pará	213.15 (142.84)	306.472 (165.333)	338.52 (186.71)
Southwest Pará	169.03 (143.70)	309.86 (253.09)	195.74*
Number of municipalities	66	35	42
2017				
Lower Amazon	125.66 (90.80)	134.82 (95.64)	134.20 (75.78)
Marajó	163.57 (62.02)	154.56 (102.01)	189.83 (64.27)
Metropolitan Belém	109.23*	289.36 (296.62)	179.90 (289.84)
Northeast Pará	185.85 (142.98)	177.03 (67.83)	123.31 (95.84)
Southeast Pará	71.63 (41.01)	216.83 (156.47)	227.87 (143.35)
Southwest Pará	-	171.01 (76.90)	232.12 (214.76)
Number of municipalities	19	48	76

Source: SIH/DATASUS.

Table 2 shows that the increase in population coverage was associated with a 22% reduction in the ACSH rate; additionally, there was a 15% reduction in hospital expenses associated with hospitalizations due to preventable conditions and an increase in the proportion of people covered by the FHS.

**Table 2:** Descriptive statistics of the study variables.

Variable	2008	2017	Difference	%Difference
FHS coverage% (teams per 3,450 people)	55.88 (31.61)	77.8 (22.72)	21.92	39.23
Hospitalizations for ACSC (per 10,000 people)	213.41 (155.16)	171.75 (135.27)	-41.66	-19.52
Hospital expenditure on ACSC (R$ per capita)	628.81* (148.46)	534.29 (281.06)	-94.52	-15.03

Source: SIH/DATASUS.

*Value deflated by the consumer price index (IBGE, October 2019).

ACSC, ambulatory care-sensitive condition; FHS, Family Health Strategy.

**Fig 2:**
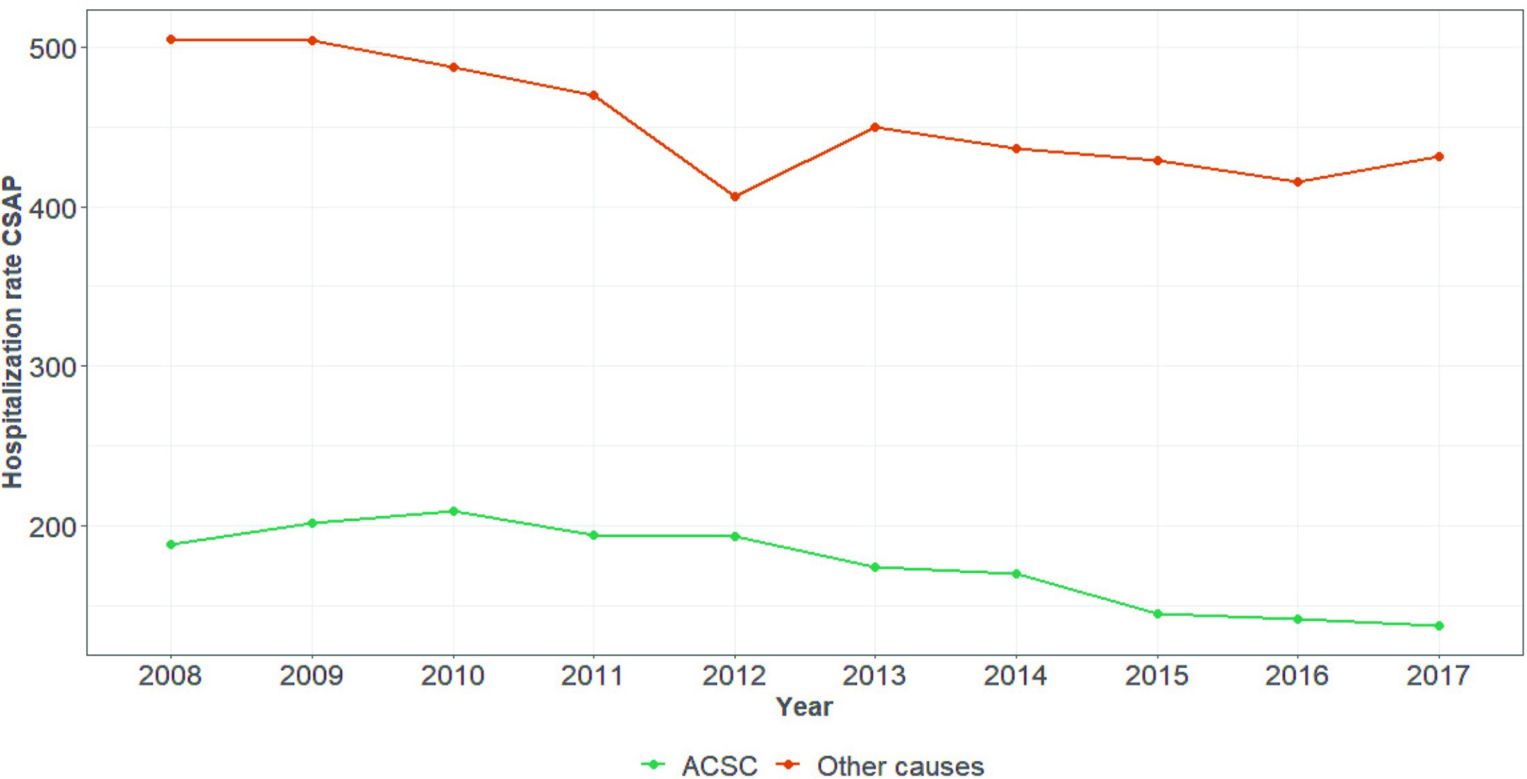
Evolution of the hospitalization rate by type of hospitalization in Pará, 2008–2017. Source: SIH/DATASUS.

Fig 2 shows that the mean hospital admission rate for other causes in the state of Pará was higher than the mean rate of ACSHs in the entire period. ACSHs showed a gradual decline, mainly after 2010, and reached a peak between 2014 and 2015.

**Fig 3:**
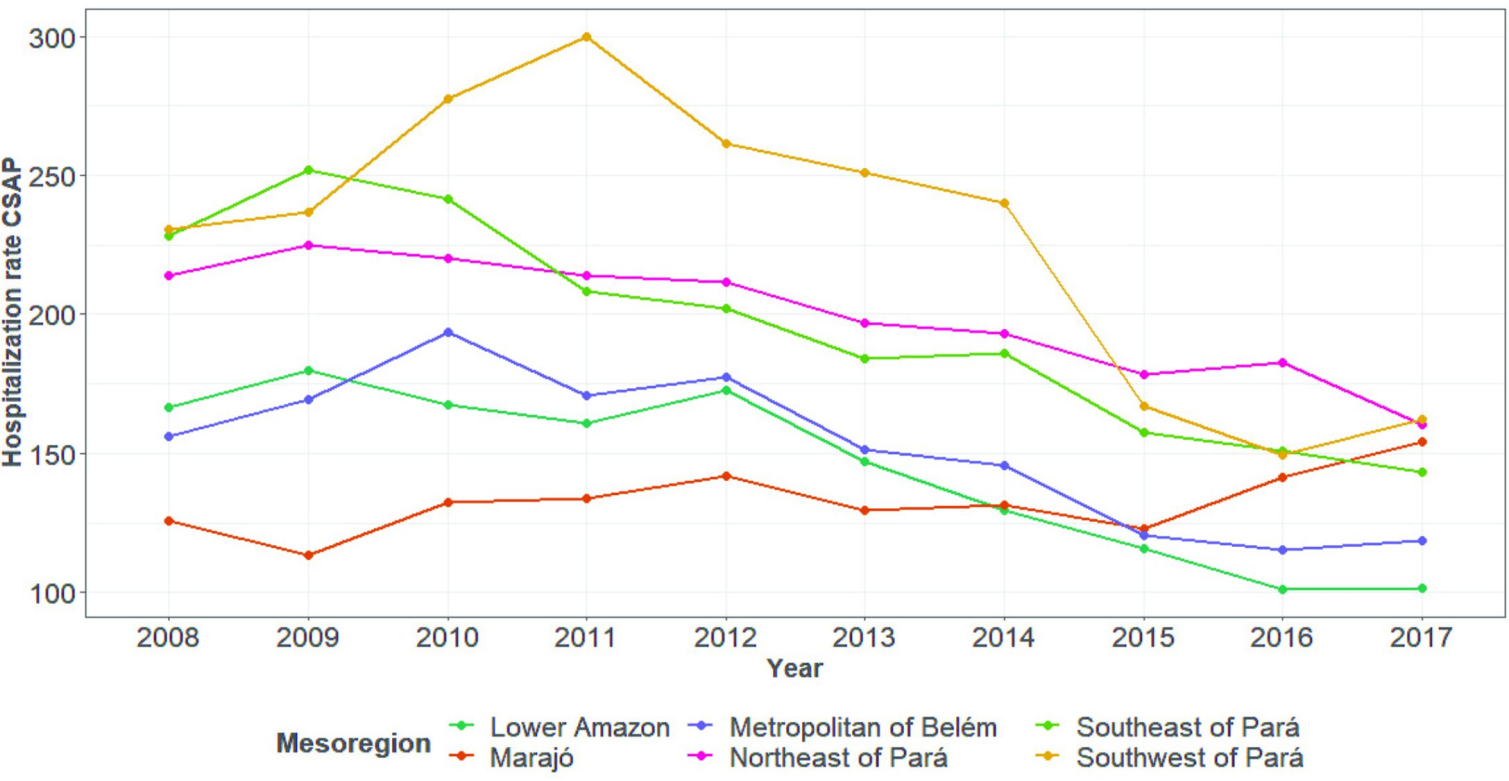
Evolution of the rate of hospitalizations for ambulatory care-sensitive conditions per mesoregion of Pará, 2008–2017. Source: SIH/DATASUS.

Fig 3 shows the evolution of the mean ACSH rate by mesoregion. A decrease occurred between 2008 and 2017 in almost all mesoregions of the state. The decrease was greatest in the Southeast Pará mesoregion during the period from 2009 to 2014, while the Marajó mesoregion had the lowest ACSH rates throughout the study period.

**Fig 4:**
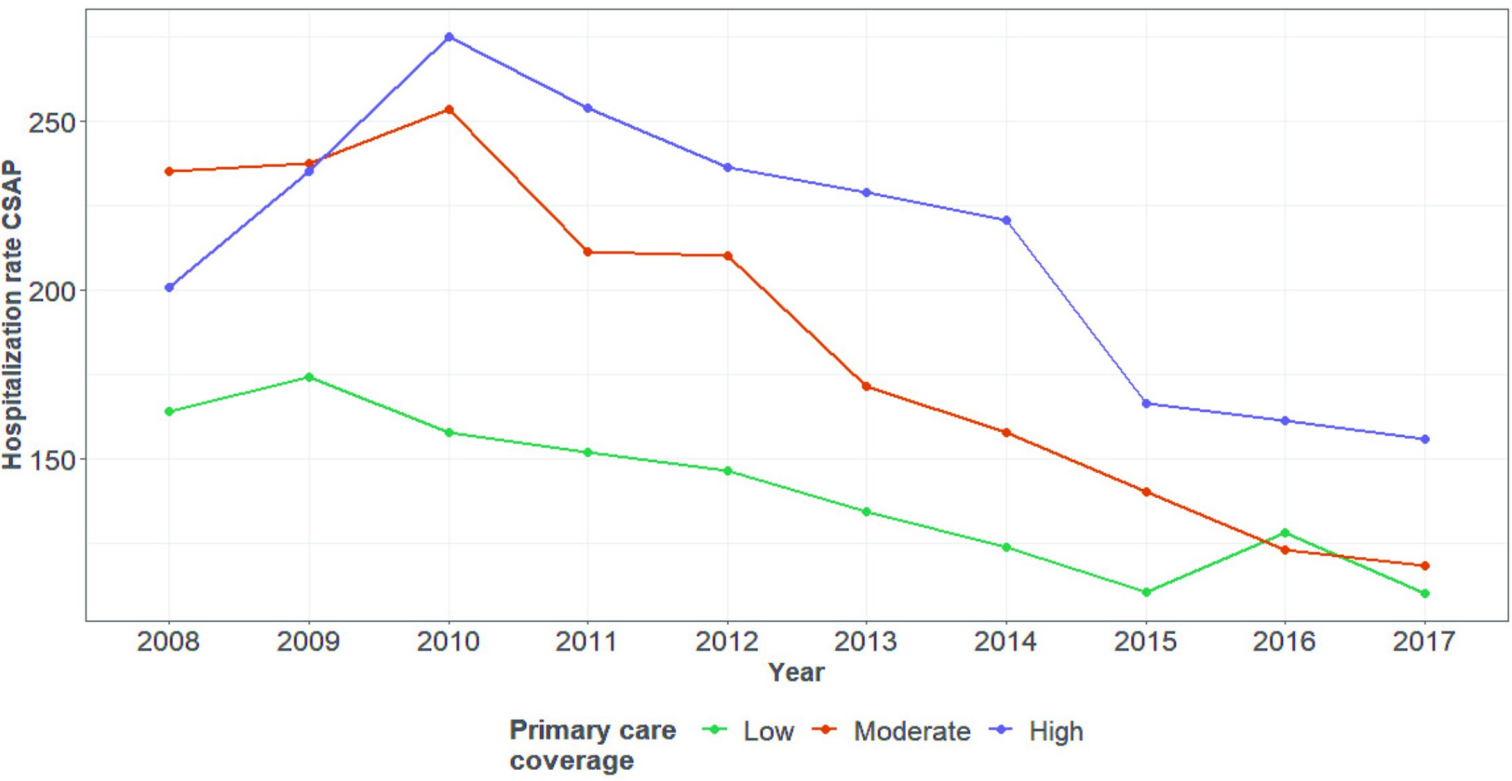
Evolution of the rate of hospitalizations for ambulatory care-sensitive conditions per coverage class in Pará, 2008–2017. Source: SIH; CNES/DATASUS.

Fig 4 shows the evolution of the mean ACSH rate in the state of Pará according to the primary care coverage class. There was a decrease throughout the period, especially in municipalities classified as having intermediate coverage. Only the Marajó mesoregion showed no decrease in this rate, and then only for municipalities classified as having low coverage (below 50%). In the Lower Amazon and Southeast Pará mesoregions, there was a reduction in hospitalization rates for all population coverage classes between 2008 and 2017.

**Fig 5:**
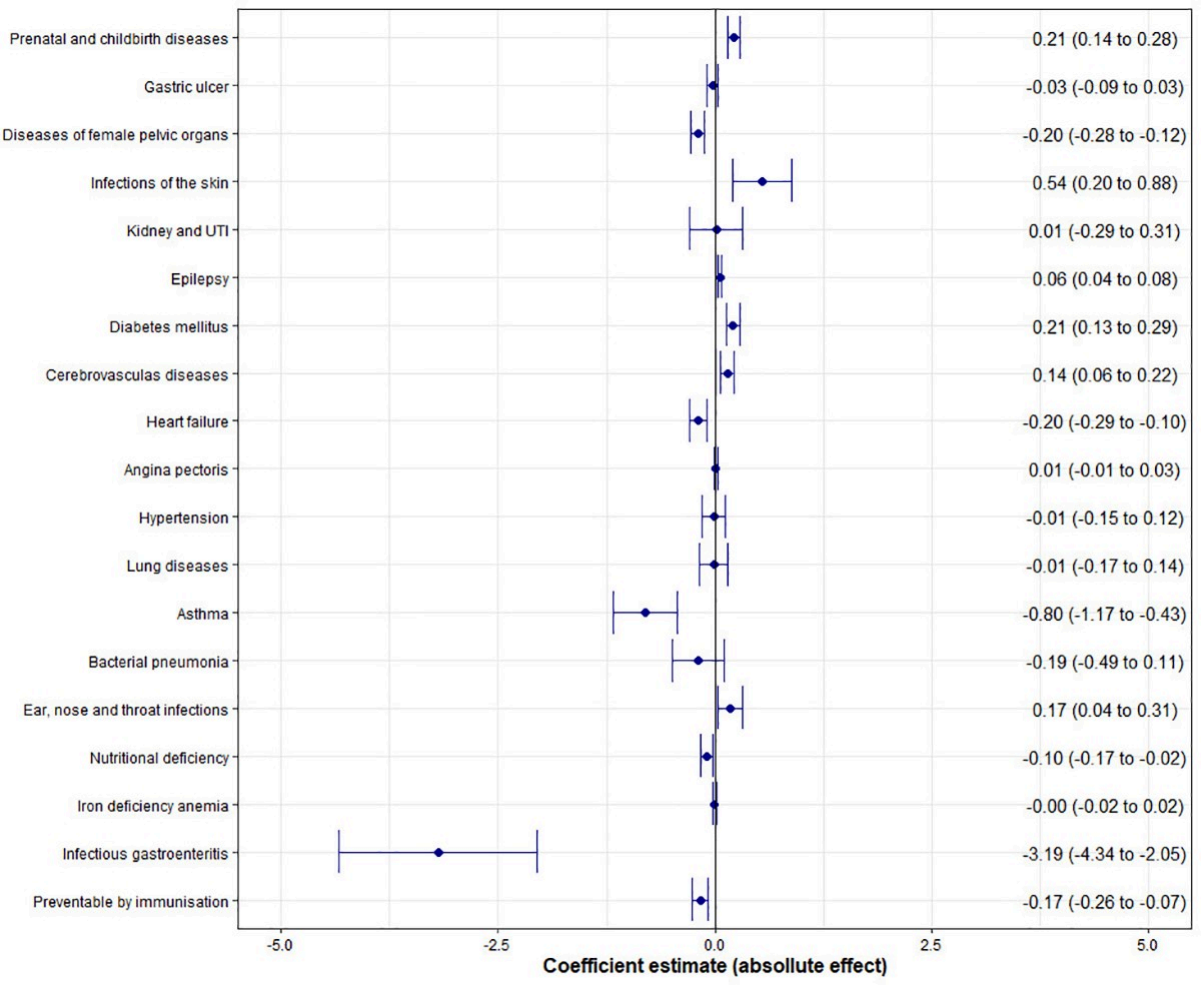
Effect of the FHS on preventable hospitalizations by disease class. Infectious gastroenteritis. Source: SIH; CNES/DATASUS.

Regarding the effect on each group according to the ICD-10 health codes included in the list of the 19 disease classes that comprise ACSHs, Fig 5 highlights the negative effect on most hospital admissions due to preventable conditions, especially admissions for infectious gastroenteritis, which accounted for 35.4% (486,781) of the total during the evaluated period and seems to have motivated the general negative trend of the sample.

We subjected the main result, the ACSH rate, to a wide range of tests. The results remained qualitatively unchanged when we used fixed effects and random effects models and when the variables controlling for economics and geography were included. Last, Table 3 shows the results of the panel regression. There was strong evidence of a reduction in preventable hospitalizations in association with FHS population coverage. The primary care expansion was associated with a decrease of 0.57 in the rate of ACSH (P<0.001) and in expenditures for preventable hospitalizations (P<0.001).

**Table 3:** Impact of the Family Health Strategy on ACSHs and hospital expenses covered by the SUS, Pará, 2008–2017.

	Population coverage		
	Model 1 (95% CI)*	Model 2 (95% CI)*	Model 3 (95% CI)*
ACSH fee (per 10,000 inhabitants)	- 0.60 (-0.93; -0.27)	-0.57 (-.89; -0.24)	-0.50 (-0.81;-0.19)
ACSH Expenditure (per 10,000 inhabitants)	- 4273.12 (-5695.99; -2850.25)	-4492.47 (-5915.96; -3068.97)	-4643.70 (-6028.57; -3258.83)

The CIs, in parentheses, are shown based on the grouping by mesoregion and weighted by the population of the municipalities.

Models are estimated by panel data modelling and include fixed effects per year.

Expenditures are constant R$ in 2017 values.

Model 1 without adjustments (without covariates); Model 2 with adjusted estimates (covariates); Model 3 with adjusted estimates (including spatial covariates).

*P<0.001.

ACSC, ambulatory care-sensitive condition; FHS, Family Health Strategy.

## Discussion

This study is pioneering in its presentation of a detailed analysis of the primary care expansion in 143 municipalities of the Brazilian Amazon. The results provide evidence of a significant reduction in preventable hospitalizations associated with a greater than 40% expansion of FHS coverage in the state of Pará between 2008 and 2017. Considering the study population, it is estimated that this rapid expansion provided medical care in isolated locations in the vast Amazon territory, thus facilitating access to and the coordination of health care.

The results corroborate previous studies that demonstrated the association between primary care expansion and the reduction in hospitalizations due to preventable causes [29–31]. Assessments of health equity in Brazil report that expanding primary health care increases the probability of access to medical care for the poorest families, especially in the North region of the country, which means that improvements in access to health for people with low and middle income levels are associated with the assigning of FHS teams [32, 33].

This study showed shown that municipalities with high and intermediate primary health care coverage rates achieved the lowest hospitalization rates. The ACSH rate was the lowest hospital admission rate during the study period and was associated with a considerable drop in hospital expenditures, thus contributing to the reduction in SUS expenditure via a more rational use of health resources.

According to estimates by the WHO, health care spending worldwide reached US$6.9 trillion in 2014; hospitals accounted for the majority (approximately two-thirds) of this spending, and 20 to 40% of resources annually are wasted due to inefficiency at this level of care [34]. Finding a balance between primary care and hospital care has been proposed to control public spending and optimize health care services [35, 36]. Through the preventive measures and monitoring of chronic conditions provided by the FHS, it is possible to reduce preventable hospitalizations so that hospitals can concentrate resources on secondary and tertiary care.

Persistent regional and social inequalities in the allocation of health care resources, as well as high medical personnel turnover and a lack of medical care, have always been causes of health inequalities in countries with marked socioeconomic differences [36, 37], especially for vulnerable populations in isolated territories of the Brazilian Amazon. To ensure the right to health guaranteed by law, 14,462 physicians were working in 3,785 municipalities in all Brazilian states one year after the start of the PMM; the largest deployment of physicians occurred in the North region of the country, with physicians comprising FHS teams in 91% of the municipalities [38]. The positive effect of the arrival of approximately 600 physicians in the state of Pará in 2013 was observed in the present study as the peak in population coverage between 2014 and 2015, which demonstrates the versatility of the FHS to adapt to the local context, expand the number of health teams and care for the population.

It is important to note that greater equity in the access to and use of health services translates into improved quality of life for the population. In the Amazonian region of Marajó, the largest fluvial-marine archipelago in the world, 60% of the population is below the poverty line [39, 40]. This region had the lowest preventable hospitalization rates after a 30% increase in primary care coverage during the analysed period. A previous study conducted in the same region showed that the expansion of FHS coverage had a positive impact on health indicators in the region [41].

The ability to expand the response to health problems that characterize the Brazilian health profile, which include high rates of chronic degenerative and infectious diseases and a high incidence of violent deaths, is a challenge for primary care, especially in North Brazil [42, 43]. Positive effects of the reduced number of hospitalizations, especially hospitalizations for infectious gastroenteritis, on the quality of life of the population were observed in this study and are directly associated with the expansion of medical care and the preventive actions provided by the FHS. Gastroenteritis is related to precarious sanitation and education conditions, especially among children and young adults, who comprise 75% of the population of the state of Pará, where sanitary sewage systems are present in only 2% of the state [44].

This study documented a considerable reduction in preventable hospitalizations, mainly for infectious gastroenteritis, with the lowest rates found in the Lower Amazonas and Southeast Pará regions. The territorial development of these regions has historically been characterized by the confrontation of problems involving isolated areas, where sustainable ways of life have butted against major power generation and hydroelectric dam construction projects, in addition to economic standards centred on mineral exploration and grain and beef cattle production. Despite claiming to be moulded around principles of sustainable development, these development policies have promoted or deepened contradictions in these regions, with harmful consequences for riverside populations and the ecosystem, and have brought misery and infectious diseases [44].

This study’s authors view strong primary care as an important component of the public system, within which the impact on the local population of improving health indicators based on the effective management of chronic and infectious conditions is evident. In addition to typical medical care, relevant actions included multidisciplinary preventive measures ranging from dietary guidance and home water treatment to oral rehydration, vaccination and clinical management of diseases with mild to moderate symptoms that can be controlled at the local health clinic, thereby avoiding flows of patients to hospitals [45]. These measures promoted the right to citizenship, improved satisfaction in the study population and reduced inequities related to universal access to health, especially in remote areas in the Lower Amazonas, Southeast Pará and Marajó regions, where there had been a severe health care shortage, thereby enhancing the living conditions of socially vulnerable populations.

Despite numerous advances, such as the reduction in preventable hospitalizations and the drop in hospital expenditure documented in this study, the SUS currently faces a series of difficulties associated with a context of an economic crisis and the austerity policies that were introduced in 2015 in the country, especially with the enactment of Constitutional Amendment 95 (EC95/PEC55/PEC241) [46, 47]. These measures included large cuts in public education and health spending, which are a cause for concern and may lead to the stagnation or deterioration of the health gains obtained via expanded primary care in Brazil, especially in regions that have long been deprived of doctors and health care, such as the vast Brazilian Amazon region.

The limitations of the present study include the fact that, despite the wide range of applied tests, the use of administrative data is susceptible to notification errors and possible failures in information processing. The analysis of expenditures using historical series has limitations as a function of inflationary variations and other economic characteristics over time. Thus, it was decided to correct the data using an economic index (IPCA/IBGE) to minimize this problem and facilitate comparisons. Moreover, the data presented here are restricted to the evaluation of the public health system. In the future, studies that use sanitation and water quality indicators may help elucidate the findings presented here; such indicators were not addressed in this study because only a small portion of the Amazonian population has access to relevant services. This study did not evaluate elements of structure (type of training of physicians, available equipment) and work process in primary care, which may have an influence on the indicators of the region.

## Conclusions

The importance of the role of the FHS in the municipalities of Pará was shown through its provision of medical care and preventive health measures, which reduced preventable hospital admissions, ensured the right to health and promoted citizenship. With the economy in free fall and the limitations imposed on health spending by a government that is little concerned with including the country’s poorest citizen, the possibility of universal health coverage established by the Brazilian constitution is at risk of collapsing. The findings of this study reinforce the need for public protection of the health of populations at risk and the positive impacts of primary health care in the Brazilian Amazon.

## Supporting information

S1 Data (XLSX)
